# Like ripples on a pond: The long-term consequences of parental separation and conflicts in childhood on adult children's self-rated health

**DOI:** 10.1016/j.ssmph.2022.101100

**Published:** 2022-04-18

**Authors:** Eva-Lisa Palmtag

**Affiliations:** Swedish Institute for Social Research, Stockholm University, Stockholm, Sweden

## Abstract

**Objective:**

The aim of the study was to explore how different forms of conflict in childhood and parental separation additively and interactively predict self-rated health (SRH) in adulthood. Furthermore, a subsample analysis investigated how different family conflicts predict SRH in adulthood within the group of separated families, controlling for post-separation circumstances.

**Background:**

Previous research shows that adult children from separated families have worse health compared to peers from intact families. Additionally, inter-parental conflicts are closely associated with parental separation and known to negatively influence children's health. Few studies have taken a broader perspective on conflicts and included conflicts beyond the inter-parental subsystem, such as within other family subsystems, into their analysis.

**Methods:**

Data were based on Swedish Level of Living Survey (LNU). Using linear probability modelling the first analysis shows that SRH in adulthood varies depending on family type and the occurrence of conflict in childhood (*n* = 6,638). The study then explored variations in SRH within separated families (*n* = 934).

**Results:**

The results show that adult children from separated families and families with different types of conflicts have worse SRH compared to their peers in intact families and families with no conflicts.

**Conclusion:**

Parental separation has long-term consequences on children's SRH and this relationship is partly predicted by parent-child conflicts.

**Implications:**

The results underline the importance of considering children's involvement when studying the separation process and its outcomes. They also suggest that interventions to help parents and children through a separation would benefit from a focus, not only on inter-parental conflicts but also, on parent-child conflicts, as well as parents' role modelling to prevent children from experiencing negative long-term health outcomes.

## Highlights

• Adults from separated families have lower well-being compared to peers from intact families.
• Family type in childhood and conflicts explain part of the differences in adults self-rated health.
• Parent-child conflicts are seldom investigated but shown to be an important explanatory variable.

## 1 Introduction

Marriages and cohabitations do not always last ‘until death do us part’ and during the last century an increased number of children experienced parental separation, both in Sweden and many other developed societies (Andersson et al., 2017).^1^ It can be assumed that children seldom have a say in their parents' decision to separate, and yet the event will change their lives in many ways. A vast amount of research has studied outcomes for children after parental separation (for reviews, see Amato, 2010, 2014; Härkönen et al., 2017). The main conclusion is that children who grow up with separated parents do, in general, worse in adulthood than their peers from intact families (Amato, 2014). On average, they have lower school grades and final educational attainment, lower psychological well-being and higher mortality risk as adults (Gähler & Palmtag, 2015; Grätz, 2015; Larson & Halfon, 2013).

Inter-parental conflict is closely associated with family dissolution, and many scholars concede that conflict, rather than parental separation per se, explains the poorer outcomes for children of separation (Harold & Murch, 2005; Ivanova & Kalmijn, 2020; Lucas et al., 2013). Rather than attempting to separate between the influence of parental separation and parental conflict, scholars have conceptualized them as two parts of a broader family dissolution process (Amato, 2010; Cox & Paley, 1997). Research on the association between parental conflict and children's life outcomes show consequences comparable to those of parental separation, such as poorer academic attainment, emotional and social behaviour problems as well as lower well-being and health (Cummings & Davies, 2002; Hanson, 1999; Harold & Sellers, 2018). Nonetheless, it is important to keep in mind that periodic conflicts also are a normal part of family life and children's socialization. As long as the child feel emotional security and the inter-parental conflicts are resolved, they will not have any harmful influence on the child (Harold & Murch, 2005). Much less attention has been given to *parent-child* conflicts (Bradford et al., 2008). In accordance with the spillover hypothesis, it could be assumed that parental conflicts and dissatisfaction in the parental relationship could spill over into other family subsystems, e.g. the parent-child relationship, thus increasing the risk of parent-child conflicts and children's negative (health) outcomes in later life (Cummings & Davies, 2002; Erel & Burman, 1995; Harold & Sellers, 2018; Low et al., 2019; Troxel & Matthews, 2004). To summarize, research that focus on the outcomes of the family dissolution process where both parental separation and different types of family conflict are included is not common. Moreover, previous research often takes on a short-term focus. Filling this research gap, this paper offers four contributions to the literature on parental separation, family conflicts and their consequences on children's health. First, the study provides an analytical investigation of the additive and interactive association that conflicts in the childhood family and parental separation might have on self-rated health (SRH) in adulthood. This addresses the question of whether or not children with separated parents have a more negative long-term experience of parental separation if they also experienced family conflict in childhood. Second, the study contributes with a differentiation between conflicts in other family subsystems than the parental one. Third, the study provides results covering long-term associations between events in childhood and children's SRH in adulthood. Finally, the study contributes to a better understanding of the heterogeneity in outcomes among adult children in separated families, by going one step further and analysing a subsample of only separated families. In this step, the study analyses whether and to what extent three common post-separation circumstances (ever lived with a stepparent, age at separation, and having weekly contact with the non-resident parent) shape the initial associations between parental separation, family conflicts and SRH in adulthood.

Health is here defined in accordance with the definition made by the World Health Organization (WHO), which defines health as “a state of complete physical, mental and social well-being and not merely the absence of disease or infirmity” (WHO, 2022). As SRH is acknowledged as global health measure that is not restrained to a predetermined health complaint, it was chosen to measure health (Idler & Cartwright, 2018). It gives a broad picture of the individual's well-being and health experience and has been validated repeatedly (Benyamini, 2011; Idler & Benyamini, 1997; Jylhä, 2009, 2010; Östberg & Nordin, 2022). Additionally, as SRH captures the physical health perception, it contributes with important knowledge as few studies estimate the association between family structure and (adult) children's physical health (Langton & Berger, 2011).

For this study, data from two cross-sectional waves (2000 and 2010) of the national representative Swedish Level of Living Survey (LNU) were used. The LNU survey contains extensive retrospective information and the study sample covers respondents born between 1925 and 1991.

## 2 Theoretical framework and previous research

### 2.1 Theoretical framework - The spillover hypothesis

In accordance with the family system approach, the family can be described as a larger overarching system that contains smaller subsystems (e.g. the parental subsystem and the parent-child subsystem) (Cox & Paley, 1997). These subsystems are hierarchically ordered and have clear boundaries with each other, but for the family to function as a whole these boundaries must be flexible so that resources and roles within the system can be exchanged (Cox & Paley, 1997). However, when boundaries and roles within these subsystems become unclear (via spillovers from conflicts or after separation and/or repartnering) it can lead to challenges and negative outcomes for the individual family members as well as the family as a whole (Cox & Paley, 1997).

Throughout the life course, the parent-child subsystem often remains one of the most central relationships in an individual's life. Therefore, the quality of this relationship also has an important role for the well-being of both generations (Thomas et al., 2017). This study draws on the spillover hypothesis to discuss how the parent-child relationship could shape the association between parental separation and inter-parental conflicts respectively and children's SRH in adulthood.

The spillover hypothesis is a leading and empirically validated (see e.g. Cummings & Davies, 2002; Harold & Sellers, 2018; Low et al., 2019) theory in the field of social research that predicts a connection between emotions in the parental subsystem and the parent-child subsystem via e.g. parenting practices and parents' attitudes (Bradford et al., 2008; Erel & Burman, 1995). It is commonly used to explain the link between parental conflicts and the parent-child relationship. It also explains any direct transfer of mood, affect or behaviour in one family subsystem to the other. In line with this, the spillover theory can predict the link between parental separation and the parent-child relationship since parental separation is a stress factor for the family members (Erel & Burman, 1995). In their extensive review, Erel and Burman (1995) discuss four mechanisms via which emotions spill over between the family subsystems. The first mechanism is labelled ‘scapegoating’ and it refers to how parents tend to focus on the child's problems or misbehaviour to avoid their own conflicts in the parental union. This can either unite the parents in their concern over the child or put the blame of their problems on the child, which decreases the parent-child relationship quality. The second mechanism focuses on social learning. The behaviour in the parental subsystem will be copied by the child, who will then use this role setting in the interaction with the parents. The influence of parental conflict on parenting behaviour is the emphasis of the third spillover mechanism. Parental conflict could cause either lacking communication about childrearing or inconsistent parenting behaviours due to disagreements on common parenting practices. Lastly, the fourth mechanism deals with family stress and how this intervenes with family subsystems. Conflicts in any of the subsystems will cause stress and lead to additional problems in each of the subsystems. Stress can also come from other factors such as chronic illness, financial problems or family transitions, which can trigger conflicts in both subsystems (Erel & Burman, 1995; see also; Brock & Lawrence, 2008; Hogendoorn et al., 2022).

To sum up, the spillover hypothesis builds on the assumption that parental conflicts and for example dissatisfaction in connection to a parental separation may transfer to the parent-child relationship. In this way hostility or disagreements in the parental relationship spill over to the parent-child relationship, which could foster parent-child conflicts and/or leaving children feeling stressed, angry, or scared. In addition, previous research has confirmed that parent-child conflict is an indirect mediator of parental conflict on children's well-being and mental health (Bradford et al., 2008). This may increase the risk for children's negative (health) outcomes later in life (Cummings & Davies, 2002; Harold & Sellers, 2018; Low et al., 2019; Troxel & Matthews, 2004). The analyses in the present paper adds to the field by showing how conflicts in different family subsystems predicts children's SRH in adulthood.

### 2.2 Previous research

#### 2.2.1 Parental separation and children's health

It is well documented that children with separated parents on average report lower levels of well-being and worse health compared to their peers in intact families (Amato, 2010; Amato & Keith, 1991; Herke et al., 2020; Langton & Berger, 2011; Raley & Sweeney, 2020). Studies showed that this association has both an immediate influence on the child's health in childhood, with outcomes that could remain over the life course (Langton & Berger, 2011; Liu & Heiland, 2009), and a gradual influence on the health with negative outcomes emerging in adulthood. The gradual influence has been shown to go via mediators that are known to predict poor health, such as lower socioeconomic status, the child's increased risk of unhealthy behaviours (e.g. smoking) and repeated stressful life changes (e.g. subsequent parental separation and/or the adult child's own separation) (Larson & Halfon, 2013; Thomas & Högnäs, 2015).

In studies using SRH and similar measurements for overall health, it has been shown that an early decline in family socioeconomic status (SES) following a parental separation (before the age of seven) is the main explanation for the long-term negative influence of a separation on SRH in adulthood (Thomas & Högnäs, 2015). As the early separation accumulates in a longer period of reduced parental resources, which then have implications for health. Additionally, it has been suggested that a decline in parental monitoring, fewer health-related investments or an increase in children's exposure to health-related risk factors after a parental separation, can lead to an increase in illness already in childhood (Langton & Berger, 2011). However, other study results instead support the conclusion that family climate such as familial cohesion and a good (or bad) parent-child relationship explained the variance in adolescents' SRH (Herke et al., 2020). The latter is in line with the theoretical mechanisms of social learning and parenting behaviour.

#### 2.2.2 Conflicts and children's health

None of the studies discussed above included conflicts in their analyses. Yet, conflict is a natural part of family life, regardless of family type. To have conflicts and to settle them successfully is an important part of children's socialization into the grown-up world (Harold & Murch, 2005; Harold & Sellers, 2018). The childhood family environment is the essential institution for children's emotional development. It is where children socialize, learn to regulate their emotions, and learn coping strategies for how to deal with difficult situations (McIntosh, 2003). Although, in cases of frequent, intense, and poorly resolved conflicts in the family, children's developmental needs are compromised. The same is true for children's physical health in childhood, with an increased risk of, for example, headaches and abdominal stress, together with an increased risk of unhealthy behaviour such as early exposure to alcohol and smoking. Consequently, conflicts in the family can have long-lasting consequences for the child's health (Harold & Sellers, 2018; McIntosh, 2003), like ripples on a pond. Since the 1950s, an increase in the frequency of conflict has been documented in Swedish childhood families. Among children born in the 1980s, one out of four reported a presence of conflict in their childhood family (Gähler & Palmtag, 2015). Research on the association between parental conflict and children's life outcomes shows consequences comparable to those of parental separation, such as, emotional and social behaviour problems as well as health problems (Cummings & Davies, 2002; Hanson, 1999; Harold & Sellers, 2018).

#### 2.2.3 The link between parental separation and parental (family) conflict and children's health (in adulthood)

Above research indicates long-term consequences from both parental separation and inter-parental conflicts on children's health, separately. Yet, these two events are often intertwined in the separation process and co-occur or follow each other in many families. In a study of short-term separation outcomes, Hanson (1999) showed that both parental divorce and parental conflict have independent associations with young children's reduced health. However, the results did not show a significant interaction effect between high level of parental conflict and separation on children's health (Hanson, 1999). In contrast, other studies concluded that family structure per se does not cause health problems among (adult)children. Instead, their results underlined that socioeconomic factors and inter-parental conflicts in childhood accounted for the largest part of the variation in children's health (Gähler, 1998; Gähler & Palmtag, 2015; Ivanova & Kalmijn, 2020; Lundberg, 1997).

Additionally, studies have shown that children in families with high-conflict inter-parental relationships experience an increase in their well-being and health after separation in terms of less illness and depression (Dronkers, 1999; Booth & Amato, 2001, for a review see Amato, 2010). However, if conflicts carry on post-separation, the improvement in well-being will be lower (Dronkers, 1999). Children from low-conflict parental relationships tend, on the contrary, to experience a decline in health if their parents separate (Amato, 2010; Booth & Amato, 2001; Feldhaus & Timm, 2015). None of these studies included child-parent conflicts so they could underestimate the potential spillover influence of parental conflict on the parent-child relationship and thus, the potential influence on children's health in the long-term. If parental conflicts are resolved by separation it could still be the case that parent-child conflicts emerge or continues post-separation.

### 2.3 Research questions

The overall conclusion drawn from previous research is that both parental separation and inter-parental conflicts have been shown to have negative influence on children's health. However, the majority of previous studies examine these two associations separately or focus on short term outcomes in childhood. In this paper, the focus is extended to cover long-term consequences for children when they reach adulthood. Therefore, the first aim is to analyse how different forms of conflict in the childhood family and parental separation additively and interactively predict self-rated health (SRH) in adulthood: Are parental separation and family conflicts associated with adult SRH? Is there an additive negative influence of parental separation *and* different family conflicts on SRH in adulthood?

Following separation new family circumstances can develop that have the potential to trigger conflict or influence family subsystems in other ways. In the second part of the study, the focus is, hence, on a subsample of respondents with separated parents *only.* Here the aim is to investigate whether, and to what extent, post-separation circumstances moderate the association between family conflicts and SRH in adulthood.

## 3 Methods

### 3.1 Data

The data used in this study originate from the Swedish Level of Living Survey (LNU). The aim of the LNU survey is to describe and measure the overall living conditions in Sweden. This makes the LNU data very rich as they cover a broad range of the respondents’ life circumstances from childhood conditions, education, family and social relations, to working conditions, and health. The first wave of LNU was conducted in 1968 and the survey has since been repeated six times (1974, 1981, 1991, 2000, 2010 and the ongoing wave from 2020/2021). LNU is a panel survey, based on a random, representative sample of approximately 1/1000 individuals from the adult Swedish population (aged 18–75 years). To ensure that the LNU survey remains representative of the Swedish population and keeps the 1/1000 proportion, each wave is updated with a refresher sample that is added to the core sample. The interviews are standardized and performed either face-to-face or via telephone. In addition to the extensive questionnaire, the LNU survey has individual register data covering e.g. area of residence, education, income and taxation. Starting in 2000, the survey was expanded to include children living in the household of the anchor respondent. All children aged 10–18 years were invited to participate in the “Child-LNU” (Swedish Institute for Social Research, 2021).

### 3.2 Sample

This study draws on data from the fifth and the sixth LNU waves, collected in 2000 and 2010. In these waves, the childhood-related questions were extended and *all* participants, including those in the panel, were asked, for example, to specify whether and between whom there were conflicts in the childhood family (up to age 16 years). The response rates were 76.6% (5,142 participants) in LNU 2000 and 60.9% (4,415 participants) in LNU 2010. In addition to the main LNU 2010 survey, there were also new interviews with the former child respondents (henceforth called Younger-LNU) who participated in the LNU child survey in 2000. The participants in Younger-LNU were aged 20–28 years in 2010 and they were interviewed with the same survey questions as the main sample. With help of a survey weight, constructed by Statistics Sweden, their data from the Younger questionnaire were added to the main sample without losing the representativity of the sample and correcting for the oversampling of young adults. The Young-LNU had a response rate of 62.6% (929 participants).

For the purpose of this study, and to accumulate the largest possible statistical power, the data from the two main waves and younger-LNU have been combined into one cross-sectional dataset. Data from panel members were only included once into the dataset and always included derived from the first wave in which the adult member participated. All the data that concern retrospective information about the childhood refers to the age period of 0–16.

The analytical sample only contains respondents who lived in an intact family or experienced a parental separation during childhood (until the age of 16 years). This excluded other family forms, such as foster families or families were the parent was single at birth as they did not experience the event of a parental separation and these family forms are likely to differ in the association with later SRH (Amato, 2010; Troxel & Matthews, 2004).

Due to missing values on the variables examined, 117 respondents were omitted. The analytical sample consists of 6,638 respondents (aged 19–75 years) of whom 5,704 had lived in an intact childhood family and 934 had grown up with separated parents.

### 3.3 Measures

#### 3.3.1 Dependent variable

*Self-rated health* (*SRH*). The dependent variable was based on the question “How do you judge your general state of health? Here it should be noted that SRH usually is measured on a four- or five-point scale though the LNU questionnaire only include the following three response categories: “Is it …” “Good”, “Bad” or “Something in-between”. This was dichotomized into *Good* (1) and *Less than good* (0 = “Bad” or “Something in-between”). It is important to underline that SRH is more than simply an indicator of ‘objective’ health (such as medical records or drug subscriptions), which it is often compared with (Idler & Cartwright, 2018). SRH conceptualizes the individual's perception of physical health, mental health, and functional limitations within a contextual, historical, subjective and objective framework (cf. p. 309 in Jylhä, 2009), even though, it is based only on a simple 1-item measure (“How do you judge your general state of health?”). To conclude, as a global measure of health, SRH is often used as an indicator that covers the individual health as a whole and fits well for the purpose of this paper.

#### 3.3.2 Independent variables

*Separated parents*. The first independent variable was based on the question: “Did you live together with both your natural (biological) parents during your whole childhood, i.e. up to age 16?”, to which the respondents included in the analyses either answered “Yes” or responded to the follow-up question “Why not?” by answering “Divorce, Judicial separation, Separation”. This was converted into a dummy variable coded 1 (yes) for ‘parental separation’ and 0 (no) for intact family during childhood.

**Conflicts in childhood.** There are three dichotomous variables measuring the respondent's experience of conflict in childhood and these are constructed from the question “Was there any serious friction in your family while you were growing up?” and the follow-up questions indicate between whom these conflicts were.

*Parental conflict.* The ‘parental conflict’ variable was coded 1 (yes) if the respondent reported serious dissensions between the parents during childhood and 0 (no) otherwise. The information comes from the follow-up question “Was there any serious friction between your biological [adoptive] parents?”. The variable was coded 1 (yes) if the respondent reported “yes” on any of the parental conflict questions.

Robustness checks have been made in the second step of the analyses and the ‘parental conflict’ variable was upgraded to also include *‘(step)parental conflict’*, based on the question “Was there any serious friction between a biological parent and a stepparent?”. The results did not deviate much from the included analysis so to keep the variable comparable to previous research results this information was left out.

*Parent-child conflict*. The ‘parent-child conflict’ variable was based on two different questions asking, “Was there any serious friction between you and your biological mother?”, and “Was there any serious friction between you and your biological father?”. The variable was coded 1 (yes) if the respondent reported “*yes*” on any of the two questions and 0 (no) otherwise.

Again, robustness checks were made and a comparison variable was constructed that also included conflicts with stepparents, “Was there any serious friction between you and your stepmother or stepfather?”. The robustness model showed an adjusted R^2^ that was lower compared to the R^2^ in the included analysis and to keep the variable comparable to previous research, the stepparent-child conflicts where excluded from the variable.

*Other conflict*. The ‘other conflict’ variable was coded 1 (yes) if the respondent reported serious friction in any other family dyad, not already mentioned.

All the conflict reports are based on retroactive questions and may be influenced by potential memory loss or the respondent's current circumstances (recall bias), but previous research on the LNU survey has shown high levels of consistency for panel respondents' answers to these questions at different time points (see Tosi & Gähler, 2016). Additionally, most of the previous studies presented earlier measure conflicts prior to separation. Yet, all the conflict reports used in this study cover the whole childhood (age 0–16) and thus, in the case of a separated family, represent the time before, during as well as after the separation. As previous research has shown that conflicts tend to increase in the first few years after a parental separation, when the parents need to (re)negotiate their responsibilities (Troxel & Matthews, 2004), these broad measures of family conflicts have the possibility to also reflect issues that follow separation.

#### 3.3.3 Control variables

The study employs a variety of controls for characteristics of the adult child as well as the family pre and post separation.

*Age* was included as a scale variable. Previous research shows that SRH depends on age, as the frequency of underlying health conditions increases (Lazarevič & Brandt, 2020; Zajacova et al., 2017). Therefore, a curvilinear specification for age (linear and quadratic age) was used in the analyses to examine whether SRH varies with age and whether the relationship fluctuates in a U-shaped fashion (‘age^2^’). *Sex* was coded 1 for *Male*. Previous studies do not find any gender difference in the reports of SRH (Lazarevič & Brandt, 2020; Zajacova et al., 2017) nor concerning children's outcomes following separation (Härkönen et al., 2017). Considering research on parental conflict, it has been shown that both sexes are similarly negatively influenced by conflicts but the nature of the consequence might differ with gender (Harold & Sellers, 2018).

*Number of siblings* was included as an indicator for the number of children that the family had to provide for and the potential risk of crowding in the household. However, having a sibling could also work as a buffer and a source of emotional security during parental divorce or conflicts (Cummings & Davies, 2002). This variable was coded 0–5, where 5 includes all respondents with five or more siblings. This categorization was made due to the skew distribution.

*Mother's education.* Previous research has repeatedly underlined that a higher maternal educational level reduces the separation disadvantages on children's well-being and mental health (Kravdal & Grundy, 2019; Mandemakers & Kalmijn, 2014). In the second step of the analysis when only adult children from separated parents are included it can be assumed that most respondents lived with their mother after separation. Almost 90 percent of all children with separated parents in Sweden were registered at their mothers' resident address in 1990s (when the youngest sample cohort were in their childhood) and alternate living was still relatively uncommon (Statistics Sweden, 2009). Considering this, the study includes a control for the mother's educational level. The variable was categorical and based on the question ‘‘which of the following best describes your mother's highest level of education?’’. Six categories were distinguished, including: missing information, no education, primary school, vocational school, lower secondary school and upper secondary school or higher, and will be included as dummy variables in the following analyses. Upper secondary school will be set as the reference category. The missing information in this variable was included as a separate dummy category, for comparison reasons, as it cannot be ruled out that the information is missing on random. Moreover, due to the smaller sample size in the second step of the analyses, where the focus was on respondents from separated parents, *Mother's education* was included as a dummy variable. Here the variable *Mother's education* was coded 1 for all mothers with an education level of upper secondary school or higher, and 0 otherwise.

*Mother born outside of Scandinavia*. In addition, to mother's education a control variable for mothers' origin was added to the analyses. Due to data restrictions, the variable was coded in two broad dummy categories, were 1 indicates if the mother was born outside of the Scandinavian countries Sweden, Norway or Denmark and 0 if she was born in Scandinavia.

*Economic hardship during childhood*. Economic hardship in childhood is negatively associated with SRH in adulthood (Granström et al., 2017; see Lundberg, 1997 for other health outcomes). It is also correlated with conflicts and could cause more tension in the family (Troxel & Matthews, 2004). The variable was coded 1 if respondents reported economic hardship during childhood and 0 otherwise. Note that, as economic hardship is known to be influenced by parental separation (Amato, 2000) and as it cannot be established whether the hardship occurred before, during and/or after the separation, robustness checks were made where economic hardship was excluded from the first stage of the analyses. The results were in general not considerably different from the results presented in Table 2, Model 4 & 5. The association between SRH and family type was about 1 percentage point larger and the R^2^ was 0.4 percentage points smaller.

#### 3.3.4 Controls for separated families only

*Lived with a stepparent.* Following a parental separation, a majority of children will live in a stepfamily setting (Harold & Murch, 2005; Turunen, 2011). Previous research has demonstrated that living in a stepfamily could influence children's emotional as well as psychological health negatively (Dronkers, 1999; Fallesen & Gähler, 2020; for girls see; Turunen, 2013). A dichotomous variable was included that indicates whether the respondent ‘ever lived with a stepparent’ during childhood (1 = yes). The data do not allow a differentiation between ever having lived with a stepfather or stepmother, respectively. Bearing in mind that most respondents lived with the mother post separation, the results may in most cases be understood as ever ‘lived with a stepfather’.

Age at *separation***.** For the respondents with separated parents, a continuous variable was included that controls for the respondent's age (0–16 years) at the parental separation. Few studies find support for the relevance of children's age at separation for their adjustment to separation (Amato, 2010). Although, a common argument is that parental separation in young ages leads to longer duration of disadvantages (post-separation) for the child (Kravdal & Grundy, 2019) while others argue that older children need more parental supervision and are more at risk for self-blame (Amato & Keith, 1991; Cummings & Davies, 2002; Kravdal & Grundy, 2019).

*Weekly contact with the non-resident parent*. A variable was added which measures the ‘frequency of contact with the non-resident parent’ following separation. The variable was coded 1 if the child met the non-resident parent at least once a week and 0 otherwise. (Robustness checks were made using the answer categories from the survey ranging from 5 “Several times a week” to 1 “Never”. Results did not deviate much from the ones reported.) Previous research has indicated that more time spent with the non-resident parent after separation is positively correlated with later SRH outcomes (Fabricius & Luecken, 2007). However, Kalmijn (2015) found that more time spent with the non-resident father increased the risk for depression for boys. Thus, previous findings are contradictory. A descriptive overview of all the variables included in the analyses are presented in Table 1.

**Table 1:** Descriptive data on the included variables, unweighted values.

Variables	Intact families	Separated families	Significance
Full sample n: 6,638	Proportion/Mean value (s.d.) n: 5,704 (86%)	Proportion/Mean value (s.d.) n: 934 (14%)	
Self-rated health, good (1)	0.76 (0.424)	0.74 (0.438)	
Parental conflict (1)	0.07 (0.250)	0.26 (0.441)	
Parent-child conflict (1)	0.02 (0.142)	0.08 (0.274)	
Other conflicts (1)	0.02 (0.127)	0.05 (0.219)	
Age (19–75/19–75)	40.78 (16.216)	31.35 (11.969)	***
Male (1)	0.51 (0.500)	0.51 (0.500)	
Mother's education (0–5)	3.00 (1.351)	3.39 (1.455)	***
Mother's education (0–1)		0.36 (0.482)	
Mother born outside Scand. (1)	0.17 (0.372)	0.11 (0.309)	
Number of siblings (0–5)	2.14 (1.436)	2.36 (1.424)	***
Eco. hardship childhood (1)	0.14 (0.343)	0.26 (0.438)	
Ever lived with a stepparent (1)		0.54 (0.498)	
Age at separation (0-16)		6.93 (4.626)	
Weekly contact with non-resident parent (1)		0.34 (0.473)	

Notes: Standard deviation (s.d.).

### 3.4 Analytical strategy

First, the descriptive data is briefly discussed, followed by an examination of the bivariate associations between parental separation and parental conflict in childhood and SRH in adulthood. Second, to answer the research questions stated above, the analysis was conducted in two stages. The first stage tested if different forms of family conflicts and parental separation in childhood additively and interactively predict self-rated health in adulthood, using weighted linear probability models (LPM). For explorative purposes, an interaction model was added to investigate if the association between parental separation and SRH depends on the occurrence of family conflicts, and vice versa. The reference category in the interaction model is intact families with no conflict. The second step of regression analyses focused on children from separated families only. In this stage, it was examined whether the possible association between different forms of family conflicts and adult SRH was shaped by other childhood circumstances related to parental separation, again using weighted LPM. Statistical significance was defined as a p-value ≤ 0.05. The estimations were produced using Stata/SE 15.1.

Note that the results should not be interpreted causally. The data cannot determine if there is, in fact, a reversed causality between SRH and parental separation and/or conflict, as there are no measures of children's health during childhood. If the child had severe health issues it could have been causing the conflicts or the separation and still be present in adulthood. Nonetheless, as previous studies using a longitudinal design have indicated that the causal direction more likely goes from the two childhood events to later health outcomes (Troxel & Matthews, 2004), it can be assumed that results presented in this study follow the same causal path.

## 4 Results

### 4.1 Descriptive results

**Fig. 1:**
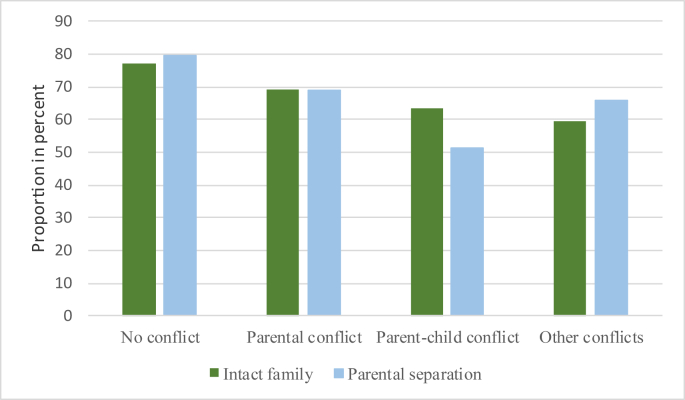
Proportion of respondents with “*Good*” self-rated health by family type, and by the presence of conflict in childhood.

Table 1, shows that 14 percent of the sample experienced a parental separation in childhood. On average, the two family types had similar SRH, where 74–76 percent of the respondents reported ‘good’ SRH. Note, however, that the average age differs substantially between the two family types, as divorce and separation rates have increased over time. This, together with increased female education levels in general, may also explain why respondents with separated parents have mothers with slightly higher education than respondents from intact families. In the group of separated families, 26 percent reported parental conflicts in childhood (30 percent when stepparents are included result not in the table) compared with only 7 percent among intact families. Parent-child conflicts (8% vs. 2%) and other conflicts (5% vs. 2%) were also more common within the group of respondents from separated childhood families. 14 percent of the respondents from intact families reported that they experienced economic hardship during childhood compared with 26 percent of the respondents with separated parents. This is in line with previous research that concluded that separated families often experience economic hardship (Amato, 2000). Table 1 also shows that among the respondents with separated parents, 54 percent lived some time with a stepparent, 34 percent had weekly contact with the non-resident parent after separation, and they were on average seven years old when their parents separated. Descriptive results in Fig. 1 show that SRH was lower, on average, among respondents that reported conflicts in childhood compared with respondents with no conflicts. The largest variance is found within the group of respondents with separated parents. Here the respondents with parent-child conflict have the lowest share of good SRH while respondents with no conflicts have the highest share. This result is not in accordance with previous research stating that health declines if a low-conflict parental relationship ends (Amato, 2010; Booth & Amato, 2001; Feldhaus & Timm, 2015).

### 4.2 SRH, parental separation and family conflicts

As presented in Table 2, Model 1 did confirm the difference in SRH between the two family types in the descriptive data in Table 1. When sociodemographic controls are included, the association between having separated parents in childhood and the SRH in adulthood was significant. This indicates that when age and gender are held constant, adult children with separated parents have worse SRH than children from intact families. Further, the results indicate that males reported better SRH compared with females. The model also shows that with the adult child's increased age, the SRH improved, yet the quadratic age term was also significant, indicating that there was a curve-linear relationship. Then again, at a closer look, comparing SRH at different ages, keeping all other variables constant (results not included), it becomes clear that the fluctuation is minor and that SRH, in general, continuous to improve with age. At the same time, the mean age of the sample is relatively low and younger cohorts are at a higher risk of having experienced a parental separation during childhood (Gähler & Palmtag, 2015), which would correlate with a lower SRH.

**Table 2:** Weighted linear probability models, dependent variable ‘SRH’ in adulthood, n = 6,638.

	Model 1 (b/robust se)	Model 2 (b/robust se)	Model 3 (b/robust se)	Model 4 (b/robust se)	Model 5 (b/robust se)
Male	0.058***	0.053***	0.053***	0.050***	0.050***
	(0.011)	(0.011)	(0.011)	(0.011)	(0.011)
Age	0.006**	0.007***	0.006***	0.008***	0.008***
	(0.002)	(0.002)	(0.002)	(0.002)	(0.002)
Age^2^	−0.000***	−0.000***	−0.000***	−0.000***	−0.000***
	(0.000)	(0.000)	(0.000)	(0.000)	(0.000)
Separated	−0.066***		−0.042*	−0.041*	−0.043*
	(0.016)		(0.017)	(0.018)	(0.018)
Parental conflict		−0.063*	−0.053*	−0.047*	−0.051+
		(0.023)	(0.023)	(0.023)	(0.029)
Parent-child conflict		−0.152***	−0.147***	−0.150***	−0.119**
		(0.040)	(0.040)	(0.040)	(0.046)
Other conflicts		−0.129**	−0.129**	−0.098*	−0.140*
		(0.046)	(0.046)	(0.046)	(0.057)
Mother's education					
Missing information				0.001	0.001
				(0.039)	(0.039)
No education				−0.091+	−0.090+
				(0.047)	(0.047)
Primary				−0.048**	−0.045**
				(0.015)	(0.015)
Vocational				−0.041*	−0.040*
				(0.019)	(0.019)
Lower secondary				−0.010	−0.009
				(0.019)	(0.019)
Upper secondary or higher (ref.)				Ref.	Ref.
Mother born outside Scand.				−0.086***	−0.086***
				(0.017)	(0.017)
Number of siblings				−0.010*	−0.010**
				(0.004)	(0.004)
Economic hardship childhood				−0.085*** (0.018)	−0.084*** (0.018)
				
Separated*Parental conflict					0.017
					(0.046)
Separated*Parent-child conflict					−0.083
					(0.067)
Separated*Other conflict					0.127
					(0.094)
**Constant**	0.753*** (0.043)	0.748*** (0.043)	0.764*** (0.043)	0.792*** (0.045)	0.795*** (0.045)
**Adj. R**^**2**^	0.063	0.070	0.071	0.091	0.091

**Notes:** + p < 0.10, *p < 0.05, **p < 0.01, ***p < 0.001.

Model 2 shows the association between SRH and different family conflicts, controlling for the basic sociodemographic variables. The results show that all the estimates for conflicts are significantly and negatively associated with SRH. The strongest of these associations seems to be between parent-child conflict and SRH.

Model 3 includes parental separation and all the different family conflict variables. The difference in SRH between children from intact families and separated families was reduced by 2.4 percentage points. The fact that both family type and the different conflict variables are significant indicates that they have an independent influence on SRH in adulthood, yet conflicts appeared to account for part of the relationship between family type and SRH (and vice versa). Furthermore, as both associations are negative and the adjusted R^2^ increased in Model 3, it can be concluded that they show an additive negative influence on SRH in adulthood. This stands against previous research that could not find any significant influence of parental separation per se on the adult child's health (Gähler, 1998; Gähler & Palmtag, 2015; Ivanova & Kalmijn, 2020; Lundberg, 1997). In Model 4, controls for mother's education, number of siblings and economic hardship in childhood were included. After this inclusion, there is a reduction in the associations between SRH and family type, parental conflict and other conflicts but no change in their directions. Among the added controls, the results show that respondents whose mother had primary education or a vocational education all had a worse SRH compared to respondents whose mother had an upper secondary education or higher. Furthermore, respondents with mothers born outside of Scandinavia had worse SRH compared to their counterparts with Scandinavian mothers. In addition, the results show that the higher the number of siblings, and if the respondent reported economic hardship in childhood, the worse the SRH in adulthood. This result is in accordance with previous studies (Granström et al., 2017; see Lundberg, 1997 for other health outcomes).

In the final model, Model 5, three interaction terms, between family type and the three conflict types respectively, were added. These terms were included to test whether the relationship between SRH in adulthood and parental separation differs depending on the occurrence of family conflicts and vice versa. None of the included interaction terms showed any significant effect. Performed *f* test statistics concluded that the included interaction terms did not add to the explain variance. The lack of a significant contribution could be due to a, for interaction modelling, relatively small sample size which results in low statistical power (Aguinis & Gottfredson, 2010). Therefore, it should be kept in mind, that even if the results indicate that the influence of parental separation on SRH is not dependent on the occurrence of family conflicts, and vice versa, an undetected interaction effect could exist in the population.

### 4.3 SRH within the group of separated families

Table 3 explores whether, and to what extent, post-separation circumstances might shape the association between family conflicts and SRH in adulthood within the group of respondents with separated parents. Model 1 includes the three different conflict variables, the basic sociodemographic controls as well as the controls from the full sample. The results stand out, as only the parent-child conflict was significantly associated with SRH. This confirms the importance of taking other types of family conflicts into the analysis to better understand the association between childhood conflicts and SRH in adulthood. Robustness checks were made, including stepparents in the conflict variables, but the results did not deviate much from the ones reported. The age of the adult child was still significant as well as the quadratic age term, but the gender association that was noted for the full sample (Table 2) was insignificant here. The lack of gender differences is in line with previous research on separation outcomes (Härkönen et al., 2017). Mother's education and mother's origin were not significantly associated with the SRH anymore but respondents who reported economic hardship had a worse SRH compared to their counterparts with no economic hardship.

**Table 3:** Separated families – weighted linear probability models, dependent variable ‘SRH’ in adulthood, n = 934.

	Model 1 (b/robust se)	Model 2 (b/robust se)
Male	0.046	0.046
	(0.030)	(0.030)
Age	0.022**	0.021**
	(0.007)	(0.007)
Age^2^	−0.000***	−0.000***
	(0.000)	(0.000)
Parental conflict	−0.036	−0.045
	(0.037)	(0.038)
Parent-child conflict	−0.232***	−0.231***
	(0.066)	(0.066)
Other conflicts	−0.012	−0.010
	(0.078)	(0.079)
Mother's education		
Upper secondary or higher	0.017	0.019
	(0.032)	(0.032)
Mother born outside Scand.	−0.041	−0.046
	(0.048)	(0.048)
Number of siblings	−0.010	−0.007
	(0.011)	(0.011)
Economic hardship childhood	−0.085*	−0.084*
	(0.037)	(0.037)
Ever lived with a stepparent		−0.054+
		(0.032)
Age at separation		0.002
		(0.004)
Weekly contact with non-resident parent		−0.004 (0.033)
**Constant**	0.504*** (0.135)	0.521*** (0.141)
**Adj. R**^**2**^	0.076	0.078

**Notes:** + p < 0.10, *p < 0.05, **p < 0.01, ***p < 0.001.

Model 2 includes the other childhood circumstances that are linked with the parental separation. However, of the included variables, none had a significant influence on the SRH in adulthood. It can be noted that the variable for ‘ever lived with a stepparent’ is significant on the 10 percent level. After computing robustness checks (model not included), it can be concluded that this variable incorporates the influence that conflicts in the stepparent subsystems have on the child's SRH. Model 2 did not change the direction of any of the previous significant coefficients. To conclude, the variables in these models explain relatively little of the total variance in SRH among children from separated families (adjusted R^2^ = 0.078).

## 5 Discussion

### 5.1 Summary and discussion

The first aim of this study was to investigate whether parental separation and different family conflicts in childhood additively and interactively predict self-rated health (SRH) in adulthood. Results show that adult children from separated families on average have worse SRH than their peers in intact families, although the association became less strong when controlling for conflicts in the family. Nonetheless, both parental separation and family conflicts had, independently, a negative influence on SRH in adulthood. This indicates that there is an additive negative consequence for adult children of separation who also experienced family conflicts. The explorative interaction model showed no evidence of any interaction effect of parental separation and family conflicts on SRH in adulthood. These results are in line with previous results from Hanson (1999) on the short-term consequence of parental separation. They also give support to the notion that separation per se does have an influence on children's health in adulthood controlling for family conflict and especially parental conflict.

To study potential heterogeneity in the outcomes after parental separation the second step of the analyses only investigated adult children with separated parents. The focus here was on post-separation circumstances and whether they shape the association between family conflicts and SRH. The results showed that none of the included controls seems to be associated with the SRH in adulthood. Furthermore, the results showed that neither conflicts between the parents, nor conflicts between others had an influence on SRH. Considering this together with the results in the first analysis, it indicates that the occurrence of inter-parental conflicts may be more associated with SRH in intact families where the conflicts might continue also after childhood. This supports the notion that a separation between the parents could lower the frequency of inter-parental conflicts and/or decrease children's risk of exposure to parental conflicts (Amato, 2010; Dronkers, 1999), which in turn decreases the risk of spillover via the four mentioned mechanisms (scapegoating, social learning, parenting behaviour and family stress) and thus the risk of worse SRH in adulthood (Erel & Burman, 1995). Parent-child conflicts had the only significant association with SRH in adulthood. This result indicates a spillover from difficulties and stress in the parental subsystem to the parent-child relationship that resulted in severe parent-child conflicts. In accordance with the social learning mechanism, the child copies the behaviour of the parents and acts as hostile within the parent-child subsystem as the parents do within the parental subsystem. Therefore, even if the inter-parental conflicts stop after a separation, the parent-child conflicts might continue. Moreover, the conflict in the parent-child system could have been even more intense if it involved the resident parent with whom the child had a daily contact. It could also indicate that conflicts where the respondent was involved, left them feeling more stressed, insecure, and guilty compared with conflicts between other family members. If the child had these feelings frequently during childhood, it increased the child's risk for long-term negative (health) outcomes (Cummings & Davies, 2002; Harold & Sellers, 2018; Low et al., 2019; Troxel & Matthews, 2004). Consequently, the results show that parent-child conflict is an important factor to consider when exploring how childhood circumstances might influence later health outcomes. This conclusion contributes with important knowledge to the field, as most previous studies that investigated the link between parental separation and children's outcomes, controlling for conflicts, only included measures of inter-parental conflicts. Therefore, they could have underestimated the importance of family conflicts on children's health throughout their life course.

### 5.2 Limitations and strengths

The study is not without limitations. As for other studies that rely on retrospective reports, this study also faces the risk of memory bias. Additionally, the study cannot rule out a possible recall bias. This occurs when respondents' memory of past happenings is influenced by current circumstances (Granström et al., 2017). Furthermore, the data did not include any information on the frequency nor on the severity of the conflicts. Parents' ability to resolve their conflicts is an important protector for children to cope and not to experience negative outcomes (Harold & Sellers, 2018). Drawing on this, it would be worthwhile in further studies to include measures of conflict frequency and parents' ability to settle disagreements. However, it can be assumed that severe and frequent conflicts as well as parental separation are critical life events, which respondents will remember well into adulthood. In addition, a selection into separation, conflicts or low SRH cannot be ruled out. This implies that the negative association between parental separation, family conflict and SRH in adulthood may be overestimated. It could be for example, that there is a selection of parent(s) into conflicts caused by their genetics. If the children inherit these traits, they might also have a more conflictual behaviour. This could explain why they associate with worse outcomes if the parents have conflicts (Harold & Sellers, 2018). Although, sibling studies have shown that genes do not explain the whole variance in children's behaviour and that the childhood environment plays an important role (Harold & Sellers, 2018). Additionally, the results from the interaction model (Model 5) should be interpreted with caution due to the small sample size. To increase the reliability of interaction modelling, further studies would benefit from a larger sample size as well as larger subgroups of separated families.

Despite its limitations, this study has several strengths. It includes three different conflict measures. As a result, the analyses highlight the importance of considering children's own conflicts when investigating the link between childhood events and health in adulthood. In addition, the study uses nationally representative survey data, which contain first-hand information on the respondent's perception of their childhood and current health status. This makes it possible to draw conclusions about important links between SRH and childhood events. Such associations could not have been examined using, for example, register-based information, as registers usually lack information about interpersonal relationships as well as first-hand information on health.

## 6 Conclusion

To conclude, both parental separation and parental conflict influence the child's SRH in adulthood. Although separation may resolve parental conflicts, these conflicts can permanently damage the parent-child bond and therefore continue to impinge on the child's health. Therefore, the results indicate, with support from the spillover hypothesis and the mechanism of social learning, that interventions aimed at helping parents, prior or post separation, to deal with conflicts could benefit from involving other subsystems of the family too. In this way, children can learn not only to cope with separation and parental conflict but also to manage their own conflicts, already in childhood. In line with the social learning theory, the interventions should support the parents and their abilities to fulfil warm and caring role models, which in turn will foster warm parent-child relationships and lower the risk of family conflicts. As parental separation rates remain high, this could help future adults to avoid long-term outcomes such as declining health.

## Ethical statement

I declare no conflicts of interest. This article is a part of a study that has been approved by the Regional Ethics Committee of Stockholm (EPN, #2009/1802-31/5).

## Author statement

The author confirms sole responsibility for the following: study conception and design, data analysis and interpretation of results, and all manuscript preparations.

## Declaration of competing interest

I declare no conflicts of interest. There are no financial conflicts of interest to disclose.

## Footnotes

1 Separation is used to denote a divorce between married parents or a separation between cohabiting parents.
